# Regulation of Cellular Metabolism by High-Risk Human Papillomaviruses

**DOI:** 10.3390/ijms19071839

**Published:** 2018-06-22

**Authors:** Imelda Martínez-Ramírez, Adela Carrillo-García, Adriana Contreras-Paredes, Elizabeth Ortiz-Sánchez, Alfredo Cruz-Gregorio, Marcela Lizano

**Affiliations:** 1 Programa de Maestría y Doctorado en Ciencias Bioquímicas, Facultad de Química, Universidad Nacional Autónoma de México, Ciudad Universitaria, Mexico City 04510, Mexico; immara02@yahoo.com.mx (I.M.-R.); cruzgalfredo@gmail.com (A.C.-G.); 2 Unidad de Investigación Biomédica en Cáncer, Instituto Nacional de Cancerología (INCan)/Instituto de Investigaciones Biomédicas, Universidad Nacional Autónoma de México (UNAM), Mexico City 14080, Mexico; adcarrillo2004@yahoo.com.mx (A.C.-G.); adrycont@yahoo.com.mx (A.C.-P.); elinfkb@yahoo.com.mx (E.O.-S.); 3 Departamento de Medicina Genómica y Toxicología Ambiental, Instituto de Investigaciones Biomédicas, Universidad Nacional Autónoma de México (UNAM), Mexico City 04510, Mexico

**Keywords:** Warburg effect, human papillomavirus, metabolism, oxidative phosphorylation

## Abstract

The alteration of glucose metabolism is one of the first biochemical characteristics associated with cancer cells since most of these cells increase glucose consumption and glycolytic rates even in the presence of oxygen, which has been called “aerobic glycolysis” or the Warburg effect. Human papillomavirus (HPV) is associated with approximately 5% of all human cancers worldwide, principally to cervical cancer. E6 and E7 are the main viral oncoproteins which are required to preserve the malignant phenotype. These viral proteins regulate the cell cycle through their interaction with tumor suppressor proteins p53 and pRB, respectively. Together with the viral proteins E5 and E2, E6 and E7 can favor the Warburg effect and contribute to radio- and chemoresistance through the increase in the activity of glycolytic enzymes, as well as the inhibition of the Krebs cycle and the respiratory chain. These processes lead to a fast production of ATP obtained by Warburg, which could help satisfy the high energy demands of cancer cells during proliferation. In this way HPV proteins could promote cancer hallmarks. However, it is also possible that during an early HPV infection, the Warburg effect could help in the achievement of an efficient viral replication.

## 1. Introduction

Cancer is a multifactorial disease involving several hallmarks. Reprogramming of energy metabolism by cancer cells causes the increase of energy intermediaries providing the cell a growth advantage associated with cell survival, migration, metastasis, and chemo/radiotherapy resistance [1,2,3]. Under physiological conditions the cells process glucose through glycolysis that generates pyruvate, which continues its metabolism in the mitochondria. On the other hand, in anaerobic conditions, glycolysis is favored with the production of lactate and consequently low concentrations of pyruvate are processed in the mitochondria [4]. Otto Warburg was the first scientist to observe that cancer cells can reprogram their glucose metabolism since, even in the presence of oxygen, the energy metabolism is favored essentially to glycolysis, a state that is called “aerobic glycolysis”, or the Warburg effect [5]. “Aerobic glycolysis” commonly increases in cancer cells, which allows them to obtain energy and metabolic intermediaries to supply the fuel needed for tumor growth [6].

Oncogenic viruses play an important role in carcinogenesis and contribute to approximately 15–20% of incident cancer cases per year [7,8]. A common feature among them is their ability to cause persistent infections that disrupt key cellular pathways. Approximately 5% of all human cancers worldwide are caused by Human papillomavirus (HPV) [9]. HPV has mainly been associated with cervical cancer, which is the fourth cause of cancer death in women worldwide [10]. HPV uses the host cell replication machinery along the viral replicative cycle to produce infectious particles; nevertheless, in some cases, the interactions of viral proteins with cell proteins induce a switch to malignant transformation, where the cells suffer a metabolic change to face their high-energy demand due to the increase in cell proliferation. This review is focused on the role of HPV proteins in the modulation of the cellular metabolism and its participation in carcinogenesis.

## 2. Cellular Energy

**Figure 1:**
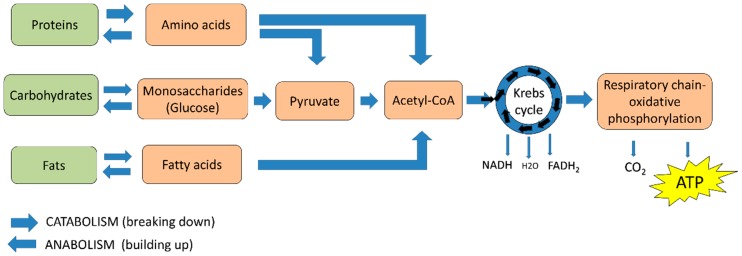
ATP production from food metabolism. Amino acids, monosaccharides, and fatty acids are produced from the metabolism of proteins, carbohydrates, and fats, respectively, from which pyruvate and/or acetyl-CoA are obtained, which, in turn, are generally metabolized in the Krebs cycle and the oxidative phosphorylation system.

Normal cells obtain their energy as adenosine triphosphate (ATP) through glucose, protein, carbohydrate, and fat metabolism, obtained from food. This process is regulated by hormones as glucagon, insulin, glucocorticoids, and growth hormone [11]. As shown in Figure 1 the metabolic intermediaries such as pyruvate and acetyl-coenzyme A (acetyl-CoA) are generated from the metabolism of amino acids, glucose, and metabolic intermediaries, such as fatty acids and glycerol. Subsequently, the production of reduced nicotinamide adenine dinucleotide (NADH), reduced flavin adenine dinucleotide (FADH_2_), as well as the high production of ATP are a result of the metabolism of acetyl-CoA in the Krebs cycle (KC), the respiratory chain, and oxidative phosphorylation (OXPHOS) [3] (Figure 1). To achieve their energy requirements, cancer cells undergo metabolic reprogramming since their metabolic needs differ from those of normal cells [12].

## 3. The Warburg Effect

**Figure 2:**
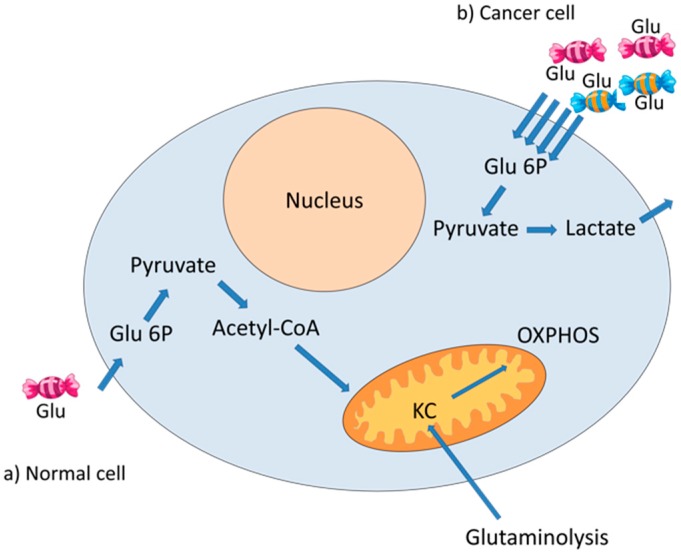
Metabolism of glucose in normal and cancer cells. The glucose (Glu) that enters the cell is phosphorylated to glucose 6 phosphate (Glu 6P) and, subsequently, is metabolized to pyruvate (**a**,**b**). In normal cells, pyruvate is metabolized to acetyl-coenzyme A (Acetyl-CoA) and continues its metabolism in the Krebs cycle (KC) and the oxidative phosphorylation (OXPHOS) system (**a**). In cancer cells, glucose entry is increased, and pyruvate is metabolized to lactate, which is then expelled from the cells (**b**). The Krebs cycle can be fed by intermediaries from glutaminolysis.

Altered glucose metabolism is one of the first biochemical features identified in cancer. Glucose metabolism provides ATP and metabolites used in a variety of anabolic pathways [13]. Most types of cancer cells increase glucose consumption and glycolytic rates. Instead of continuing its metabolism through the Krebs cycle, most of the pyruvate produced in cancer cells is converted to lactate by lactate dehydrogenase (LDH); subsequently, the lactate is secreted by the muscle into the blood to be transformed back into glucose in the liver by the Cori cycle; otherwise, lactate can immediately replace glucose as a fuel for almost all cells in the body that contain mitochondria [14]. This metabolic modification occurs even when there are enough oxygen molecules for mitochondrial respiration and the integrity of the mitochondria is optimal [15]. It is not clear why cancer cells process pyruvate into lactate instead of entering it into the Krebs cycle. Apparently, cancer cells require glycolytic intermediaries, generated by the Warburg effect, to meet anabolic needs and to promote their growth and long-term maintenance [16]. Nevertheless, cancer cells can also obtain energy from a smaller fraction of glucose that is metabolized through the Krebs cycle. Moreover, cancer cells use anaplerotic pathways to obtain energy, such as the glutaminolysis, which also feeds the Krebs cycle [17] (Figure 2).

## 4. Human Papillomavirus

A persistent infection with HPV is the main risk factor for the development of cervical cancer [18]. More than 200 HPV types have been identified [19] and 40 of them are associated to anogenital cancer. HPV infections are sexually transmitted and according to their oncogenic potential and prevalence in cancer are classified in low-risk HPVs (LR-HPV) and high-risk HPVs (HR-HPV) [20]. HPV types 16 and 18 are the most frequently found in cervical cancer, in 60% and 15% of the cases, respectively [21]. Moreover, HR-HPV are related to other cancers as vulvar, vaginal, anal, penile [22], and oropharyngeal squamous cell carcinoma [23].

### 4.1. HPV Genome

HPVs are small non-enveloped viruses with a double-stranded circular DNA with a length of about 8 Kb. A fragment of the HPV genome of approximately 900 bases, named the long control region (LCR), regulates viral replication and transcription. The HPV genome encodes the E1, E2, E4, E5, E6, and E7 proteins, which are early expressed during the viral infection. L1 and L2 proteins are expressed later during the productive infection and constitute the viral capsid [24]. HPV replication depends upon the host cell differentiation program [25]. *E1* and *E2* are expressed at the beginning of the infection, bind to the LCR and participate in viral replication and transcription. E4 is expressed mainly during later stages of infection, contributes to the amplification of the viral genome and disrupts the cellular keratin network, facilitating virus release [26]. E5 inhibits maturation of endocytic vesicles from early to late endosomes [27]. E6 and E7 are the main viral oncoproteins, that as multifunctional proteins, regulate the cell cycle through their interaction with a set of cellular proteins, being within the most relevant the tumor suppressor proteins p53 and pRB, respectively [28,29]. E6 and E7 oncoproteins target numerous cellular proteins and thus promote cell cycle progression, apoptosis inhibition, DNA damage and evasion of the host immune response [30]. 

For example, the PDZ-binding motif (PBM) of E6 oncoproteins interacts with PDZ domain-containing proteins, such as disk large homolog 1 (Dlg1), Scribble, and membrane-associated guanylate kinase (MAGI)-1, -2, -3, which, in many cases, lead to their proteasome-mediated degradation [30]. High-risk E6 oncoproteins interact with at least 14 identified cellular PDZ-containing proteins, whose degradation gives rise to changes in cell morphology, reorganization of microfilament network and loss of tight junctions, associated with the immortalization of keratinocytes [30,31].

E7 is the main transforming protein of HR-HPV due to its interactions with at least 20 protein partners including pRb, c-Jun, and multiple epigenetic elements such as histone deacetylase 1 (HDAC1), DNA methyltransferase (DNMT1), Enhancer of zeste homolog 2 (EZH2), and CBP/p300 [32]. Dimerization of E7 with CBP/p300 provokes the acetylation of pRb, causing cell cycle disruption [33].

Moreover, it has been demonstrated that E6 and E7 oncoproteins from HPV16 impair the function of diverse enzymes involved in the homologous-recombination pathway reducing its ability to complete the double-strand break (DSB) repair, which contributes to genomic instability [34].

### 4.2. HPV Replicative Cycle

The HPV replicative cycle begins in the basal layer of the squamous epithelia, where viral particles arrive through micro wounds and there they complete their life cycle, taking advantage of the differentiation program of the host epithelial cells [25]. After the viral entry, the HPV genome is replicated as an episome. At the initial infection in undifferentiated basal cells, low levels of the E1, E2, E6, and E7 proteins are expressed, delaying normal keratinocyte differentiation. E1 is the only viral protein with enzymatic activity: it is a helicase/ATPase which is recruited by E2 at the origin of viral replication, generating approximately 50 to 100 copies of viral episomes per cell [35]. These numbers increase to thousands of DNA viral copies throughout the differentiation of the epithelium [36]. From the middle to the upper differentiating epithelial layers, E6 and E7 are expressed in high amounts [37] and the viral life cycle is completed by the expression of L1 and L2 proteins in the uppermost layer of the epithelium. Finally, the viral genome is encapsidated and the mature virions are released [35]. A complete viral life cycle does not represent a risk for cancer development. Moreover, near 90% of HPV infections, even with the high-risk types, are eliminated within two years [38]. A persistent infection with HR-HPV is the main determinant for cervical cancer development; where the accumulation of damaged DNA, probably due to the interactions of E7 and E6 with their cellular targets, leads to genomic instability and, in most cases, to the integration of the viral genome into the host genome [39]. Viral genome integration precedes the overregulation of *E6* and *E7* oncogenes, since in most of the cases *E2* gene is disrupted, which causes the overexpression of *E6* and *E7* oncogenes [40]. It has been reported that the ratio of HPV integrated genome copies in relation to DNA episomal particles increases as the cervical lesion progresses to cancer [41]. Nevertheless, there are other possible mechanisms of E6/E7 deregulation [40]. For example, in some cases, HPV DNA copies are found integrated in tandem and, even when the *E2* gene is not broken, the E2 binding sites in the LCR are found methylated, which affects the binding of E2, avoiding its repressive effect [42]**.** Tandem integration of the HPV genome can also cause the formation of super-enhancer like elements which strongly activate *E6*/*E7* expression [43].

## 5. HPV Oncoproteins and Cellular Metabolism

**Figure 3:**
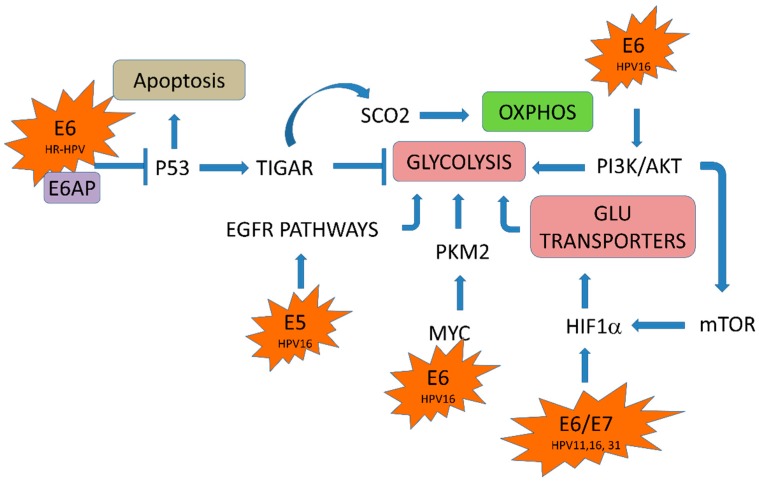
Modulation of the Warburg effect by HPV oncoproteins. E6, E7, and E5 viral proteins activate the glycolytic pathway (arrows). E6 with E6 associated protein (E6AP) inhibits repressors of the glycolytic pathway such as p53, which activates the TP53 induced glycolysis and apoptosis regulator (TIGAR), a glycolytic repressor protein (truncated arrow). TIGAR activates cytochrome c oxidase assembly protein (SCO2), activator of oxidative phosphorylation (OXPHOS). Hence, the absence of p53 promotes glycolysis. E5 activates epidermal grow factor receptor (EGFR) pathways; E6 activates Pyruvate kinase isoform 2 (PKM2), PI3K/AKT and rapamycin complex 1 (mTOR); E6 and E7 activate hypoxia-inducible factor (HIF1. Glu: glucose). The HPV type is indicated.

The continuous expression of E6 and E7 are required to maintain the malignant phenotype [44,45,46]. Accumulating evidence suggests that HPV is involved in the reprogrammed metabolism of cervical cancer cells. Diverse interactions of E6 and E7 with cellular partners have been demonstrated, and many of those affect cell biological functions, leading to transformation. E6 interacts with the E6 associated protein (E6AP), an E3 ubiquitin ligase, targeting cellular proteins to be ubiquitinated for proteasomal degradation [47]. In this way HR-HPV E6 induces p53 degradation by forming a complex with E6AP [48] and, thus, p53 dependent apoptosis is blocked [49]. It has been reported that p53 regulates genes involved in metabolic processes, such as glycolysis and OXPHOS pathways, which are inhibited and activated by p53, respectively [50]. For instance, p53 induces the expression of the TP53-induced glycolysis and apoptosis regulator (TIGAR), a transcriptional factor that inhibits glycolysis. p53 also upregulates the cytochrome c oxidase assembly protein (SCO2), necessary for the assembly of the complex IV of the electron transport chain [51]. Therefore, HR-HPV E6 modulates metabolic pathways due, in part, to its interaction with p53 (Figure 3). E6 HPV16 interacts with c-Myc which is a transcription factor that promotes the expression of genes involved in the control of glycolysis, such as enolase A, hexokinase II, lactate dehydrogenase A, phosphofructokinase and glucose transporter [52,53]. Moreover, E6 also activates the mammalian target of the rapamycin complex 1 (mTORC1) pathway mediating the increase in protein kinase B (PKB, also known as Akt) activity through the phosphoinositide-dependent kinase-1 (PDK1) and mTORC2 pathways. mTORC1 signaling cascade serves as a metabolic sensor that responds to nutrient and growth factor availability and leads to accumulation of hypoxia-inducible factor 1 (HIF1), a transcription factor involved in the cellular adaptation to hypoxia and other hypoxia response elements regulated proteins, such as the glucose transporter 1 (GLUT1) [54,55]. Therefore, HPV16 E6 can also regulate glycolysis by its association with c-Myc and PI3K/AKT pathways (Figure 3).

The E7 oncoprotein can also promote the glycolytic pathway by modulating HIF1. Rodolico et al. (2011) found a strong association between the expression of HPV16 E7 and HIF1 in oral squamous cell carcinoma in patients infected with HPV16 [56]. HIF1 has two subunits of which HIF1α is regulated by oxygen concentrations and dimerizes with HIF1β to form the transcription factor HIF1. HIF1 expression also enhances the transcription of genes involved in glucose metabolism [57]. The activation of HIF1α contributes to the Warburg effect through the upregulation of glycolysis and downregulation of oxidative phosphorylation [58] (Figure 3). On the other hand, the E5 oncoprotein from HPV16 could indirectly regulate the Warburg effect through the axis of the epidermal growth factor and its receptor (EGF–EGFR). E5 stimulates EGFR signaling pathways, promoting a prolonged activation of the extracellular signal-regulated kinases-1,2 (ERK1/2) and AKT in response to EGF [59]. It has been shown that EGFR pathway promotes an enhancement of the glycolytic metabolic program in oral cancer cells [60].

### 5.1. HPV E6/E7 Oncoproteins and Glucose Transporters

Most of the nutrients which are necessary for cells to synthesize macromolecules and carbon sources are hydrophilic and do not diffuse freely through the cell double layer membrane, requiring help for their entry into the cell. For instance, glucose enters the cell through two families of glucose transporters, the GLUT transporters (GLUTs) and SGLT Na+/glucose cotransporters (SGLT1 and SGLT2 in epithelial cells) [61,62,63].

GLUTs are membrane proteins that transport monosaccharides, polyols, and other small carbon compounds [64]. GLUT transporter family internalizes glucose by a mechanism of facilitated diffusion. In cancer cells, there is an upregulation of specific glucose transporters in the plasma membrane. Under hypoxia or nutrient deprivation, tumor cells overexpress at least one of the GLUT transporters, predominantly GLUT1. A high expression of this glucose transporter has been described in many types of cancer including cervical cancer [65,66]. It has been reported that GLUT1 is a transcriptional target of HIF1, which activates a panel of target genes for example GLUT-1, GLUT-3, carbonic anhydrase IX (CAIX), and vascular endothelial growth factor (VEGF), promoting the transcription of glycolytic enzymes [58,66,67]. Cancer cells have a high demand of nutrients due to their high rate of proliferation, so the entry of glucose by facilitated diffusion through GLUT transporters could be insufficient, being required other mechanisms of glucose uptake may be required. For example, SGLT Na+/glucose cotransporters internalize the glucose in the cell through a secondary active transport coupled to Na+ entry, mediated by the Na+/K+ ATPase. The energy produced by the consumption of glucose is higher than that required by the Na+/K+ ATPase [68]. SGLT1 which is present in HPV-positive cervical carcinoma cells helps cells to accumulate glucose even when there is a low concentration of extracellular glucose [63]. In addition, in several tumors EGFR is highly expressed and this receptor is physically associated with SGLT1, inducing its stabilization and promoting glucose entry into cancer cells [64,65,66,69].

The expression of GLUT1 is under the control of multiple transcriptional factors, one of which is p53 that directly represses the transcription of GLUT1 and GLUT4 and indirectly GLUT3, limiting glucose uptake in cancer cells, which slows down their growth. Therefore, the degradation of p53 mediated by HPV E6 oncoprotein could lead to overexpression of GLUT1 and to elevation of glucose uptake in cervical cancer cells [70].

It has been reported that HPV16 E6 contributes to the Warburg effect preventing the interaction and degradation of HIF1 by Von Hippel-Lindau (VHL) tumor suppressor [71]. Moreover, it has been proposed that HPV E6 and E7 increase GLUT1 expression through the upregulation of HIF1α in lung cancer [72]. Interestingly, in Xenopus oocytes expressing SGLT1 it was found that the co-expression of HPV18 E6 significantly increased the SGLT1 protein abundance in the cell membrane [68].

### 5.2. HPV in the Glycolytic Pathway

**Figure 4:**
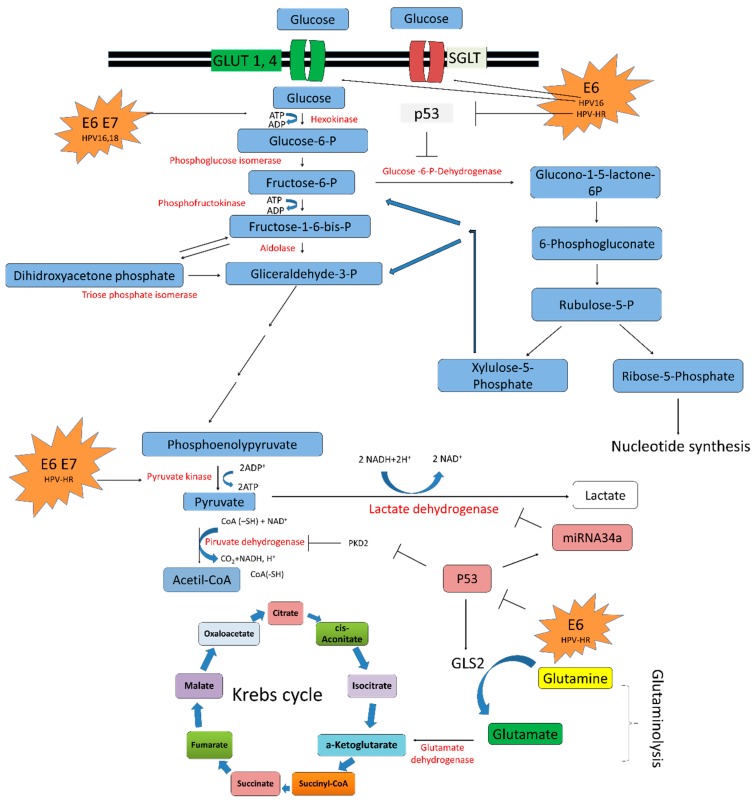
The modulation of glucose metabolism by HPV oncoproteins favor glycolysis instead of OXPHOS. HR-HPV E6 oncoproteins promote glucose uptake through the overexpression of glucose transporters GLUT and SGLT. Both E6 and E7 oncoproteins induce the expression of hexokinase, enzyme associated to the first step of glucose metabolism. Moreover, E6 can promote nucleotide synthesis through the degradation of p53, which is an antagonist of glucose 6-P-dehidrogenase, a key enzyme in the pentose phosphate pathway (PPP). The glycolytic pathway is carried out in two phases. First, in the preparatory or glucose activation phase, a six-carbon glucose molecule breaks down into two molecules with three carbons each: one glyceraldehyde-3-phosphate and the other is dihydroxyacetone phosphate, which is transformed into glyceraldehyde-3-phosphate. In these reactions, two molecules of ATP are consumed. Second, the energy extraction phase, involves the conversion of the two glyceraldehyde-3-phosphate molecules into two pyruvate molecules by pyruvate kinase, which is activated by E6 and E7 oncoproteins. This process results in the production of four ATP molecules through substrate-level phosphorylation. Pyruvate is metabolized to acetyl-CoA by pyruvate dehydrogenase, which, in turn, is negatively regulated by pyruvate dehydrogenase kinase 2 (PDK2). PDK2 is activated in the absence of p53, a process induced by E6, avoiding acetyl-CoA production. Moreover, p53 degradation avoids glutaminase 2 (GLS2) activation and glutaminolysis, which in turn decreases α-ketoglutarate levels. Thus, E6 does not permit an optimal function of the Krebs cycle, inducing lactate production by lactate dehydrogenase. E6 prevents the expression of miRNA34a and lactate dehydrogenase deactivation. HPV type is indicated. T arrows indicate inhibition. Black line arrows and thick blue arrows indicate the direction of the reaction.

Glycolysis also named as the Embden-Meyerhof pathway, is the first step of the glucose metabolism. It covers ten reactions that occur in the cytosol, where two molecules of pyruvate are obtained from the metabolism of one molecule of glucose [4] (Figure 4).

Three key enzymes participate in glycolysis mediating irreversible reactions: hexokinase (HK), phosphofructokinase-1 (PFK-1) and pyruvate kinase (PK). In aerobic conditions the pyruvate is oxidized in the Krebs cycle into acetyl-coA and then into CO_2_ and H_2_O. Meanwhile, under anaerobic conditions pyruvate can follow the fermentation pathway and produce lactate [4,73]. Finally, the total energy in form of ATP produced in aerobic conditions consists of 36 ATP molecules, in contrast to anaerobic conditions which accumulates only two ATP molecules (Figure 4).

The enzyme hexokinase 2 (HK2) is an important regulatory protein of the “Warburg effect”. In most normal tissues, it remains at very low levels but it is found frequently elevated in cancer [74,75]. The increased glycolysis in cancer cells promoted by the rise of HK2, provides energy and also precursors important for tumor growth [6]. In addition, HK2 supplies intermediaries for the Krebs cycle through the use of glutamine derived carbon in anaplerosis pathway [76]. When the cell is deficient in glucose, HK2 facilities autophagy by maintaining cell energy in homeostasis [77].

Zeng Q et al., in 2017 [78] reported that in mouse embryo fibroblasts, HK2 and consequently glycolysis, were increased in a c-Myc-dependent manner when HPV16 E6/E7 were ectopically overexpressed [79]. This suggests that E6/E7 oncoproteins directly activate the expression of HK2 and reprogram the HPV transformed cells to glycolysis as a new strategy to maintain the transformed phenotype [78,79]. Furthermore, it was demonstrated that E6/E7 silencing in the HeLa cell line caused a strong decrease of HK2 at mRNA and protein levels, mediated in part by the decrease in c-Myc expression and the increase in miR-143-3p [80].

Other evidence related to HPV in reprogrammed metabolism of cervical cancer cells is the interaction with pyruvate kinase (PK) protein (Figure 4). The PK protein has two isoforms, an embryological isoform (M2) with elevated enzymatic activity, and an adult isoform (M1) [81]. In NIH 3T3 cells, HPV-16 E7 oncoprotein directly binds to M2-PK inducing its dimerization, which promotes nucleic acid synthesis and cell proliferation [82]. In addition, malignant transformation of cells by HR-HPV E6 and E7 oncogenes induced a PK isoform switch from M1 to M2. This switch causes a shift from normal cellular metabolism to elevated glycolysis, beneficial for tumor growth [83].

The lactate dehydrogenase A (LDHA) enzyme is responsible for the processing of pyruvate to lactate in the absence or in low levels of oxygen. Recent studies revealed that microRNAs (miRNA) are involved in regulation of the Warburg effect by targeting specific enzymes [84,85]. miR-34a is a tumor suppressor upregulated by p53 [86]. Zhang et al. (2016) showed that miR-34a inhibited the lactate production by directly targeting LDHA. In cervical cancer, reduced levels of miR-34a has been reported as a result of p53 degradation by E6 [87]; therefore, metabolic reprogramming mediated by the absence of miR-34a could play an important role in cervical carcinogenesis (Figure 4).

### 5.3. HPV in the Krebs Cycle

p53 induces the expression of proteins, such as the cytochrome c oxidase 2 (SCO2) [51] (Figure 3), the apoptosis-inducing factor (AIF) [88] and ferredoxin reductase (FDXR) [89], which are associated with the maintenance of the mitochondrial integrity and oxidative phosphorylation. This transcription factor also induces OXPHOS promoting the inhibition of pyruvate dehydrogenase kinase 2 (PDK2), which is an enzyme that deactivates the pyruvate dehydrogenase complex (PDC) [90] (Figure 4). Thus, PDK2 deactivation induces the production of acetyl-CoA by PDC, an essential molecule in KC. Furthermore, p53 has a very important role in the glutamine metabolism pathway, which is an alternative via that feeds KC, since p53 induces the expression of glutaminase 2 (GLS2), an enzyme that converts glutamine to glutamate, which, in turn, is changed into α-ketoglutarate supplying the KC [91] (Figure 4). Thus, p53 has a crucial role in rising the KC and OXPHOS. However, in the presence of HR-HPV E6, p53 is degraded via proteasome and the conditions for the Warburg effect are then favorable (Figure 4).

### 5.4. HPV E2 Protein and the Oxidative Phosphorylation System

**Figure 5:**
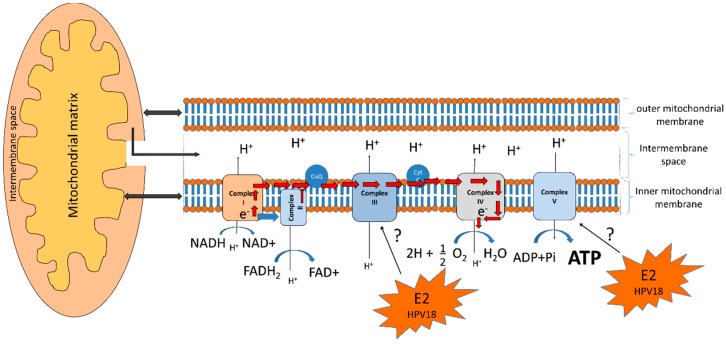
Modulation of the oxidative phosphorylation system by HPV18 E2 protein. Electrons are transferred from NADH or FADH_2_ to O_2_ using a series of electron carriers: Complex I, II, III, IV, and V favor the translocation of protons and generate an electrochemical gradient of protons in the intermembrane space. Red arrows show the electron flux. Electrons (e^−^); protons (H^+^); oxygen (O_2_); water (H_2_O); coenzyme Q (CoQ), and cytochrome C (Cyt C). Since E2 modifies the mitochondrial cristae morphology, releasing ROS, it could possibly modulate Complex III, which is a mediator of mitochondrial ROS production; and it could also modulate Complex V, which is a regulator of the mitochondrial cristae structure. The question mark (?) indicates the possible effect of E2 on the complexes III and V.

The OXPHOS system occurs in the mitochondrial inner membrane and consists of five enzyme complexes. The first four complexes: Complex I or NADH dehydrogenase, Complex II, or succinate dehydrogenase, Complex III, or ubiquinol-cytochrome *c* oxidoreductase, and Complex IV, or cytochrome, along with two electron carriers: ubiquinol and cytochrome C, form the electron transport chain which generates a proton gradient used by Complex V, or ATP synthase, to generate the majority of cellular ATP [92] (Figure 5).

In aerobic conditions, 36 ATP molecules are produced from the complete oxidation of glucose into CO_2_ and H_2_O. It has been shown that mitochondrial respiration is also a major source of reactive oxygen species (ROS) that damage the cell and are markers of OXPHOS dysfunction [93] (Figure 5).

The E2 proteins from HR-HPV are negative regulators of E6 and E7 oncoproteins. E2 proteins actively shuttle their location between the cytoplasm and nucleus. In the cytoplasm, E2 can promote apoptosis [94] and, in the nucleus, it induces chromosomal instability and DNA breaks during mitosis which possibly facilitates the integration of the HPV genome into the cellular genome [95]. Lai et al., 2013 determined that E2 can also be found in the mitochondrial membrane where it modifies the cristae morphology increasing mitochondrial release of ROS and, therefore, modifying cellular respiration, which correlates with HIF1 stabilization and increased glycolysis [93]. As proteins from Complex III are central mediators of mitochondrial ROS production [96] and ATP synthase is a regulator of the cristae structure [97], E2 could modulate the mitochondrial structure and ROS release through the interaction with those proteins (Figure 5). During cellular homeostasis ROS are used as second messengers that influence cell proliferation and differentiation; however, their increase due to mitochondria alteration, or to processes such as the Warburg effect, could produce oxidative stress (OS) and, consequently, damage to DNA, lipids, or proteins. Cells have a battery of antioxidants, such as glutathione (GSH), superoxide dismutase (SOD) 1, 2 and 3, catalase (CAT), peroxidases (Prxs), and thioredoxins (Trxs), among others, which respond quickly and efficiently against OS [98]. It has been shown that the co-expression of E2 and E1 from HPV18 decreases GSH levels and SOD1 and 2 activity inducing an increase in ROS levels and DNA damage [99].

## 6. The Warburg Effect in the HPV Replicative Cycle

It has been proposed that the Warburg effect represents a metabolic strategy that allows cancer cells to optimally meet energy demands posed by stochastic or fluctuating tumor environments [100], so that oxidative phosphorylation and aerobic glycolysis work in a complementary way to satisfy ATP demands as required. Even though OXPHOS yields a maximal number of ATP molecules, its production is too slow for satisfying peaks of fluctuating ATP demands; in contrast, although aerobic glycolysis is less efficient, it can produce ATP with a very fast flux, more than 100-fold faster than oxidative phosphorylation [101]. Therefore, when tumor cells require fast accessibility to ATP, in order to temporarily face increased short-term energy demands, as in the case of proliferation [102], migration [103], or invasion [104], the energy production is optimized by glycolysis, which, in general, can represent a normal physiological function [105], which is increased in cancer cells.

In such a way, HPV-related cancer would benefit from the Warburg effect to maintain cancer hallmarks and it has been demonstrated that different interactions of HPV proteins with cellular proteins promote the Warburg effect. Now, the question arises whether the Warburg effect could also favor the HPV replicative cycle during the infectious process.

In HPV infected cells, a peak of ATP could be demanded for the synthesis of viral DNA during HPV replication. This process needs high amounts of energy in the form of ATP, which could be rapidly obtained from increased glycolysis, to be used by the E1-helicase/ATPase viral protein for DNA uncoiling and for the stimulation of DNA synthesis through the recruitment of cellular replicative DNA polymerases, which is dependent on the ability of the E1 helicase to hydrolyze ATP [106]. Replication of HPV also requires high amounts of nucleotides, which could be obtained from the processing of a glycolytic intermediary, the glucose-6-phospate (G6P), which is a substrate of the glucose-6-phosphate dehydrogenase (G6PD) in the pentose phosphate pathway (PPP) [13]. G6PD is a rate-limiting enzyme for PPP, which produce ribose and nicotinamide adenine dinucleotide phosphate (NADPH) (Figure 3). Ribose is an essential molecule in the synthesis of nucleotides, while NADPH is a cellular antioxidant and coenzyme of multiple cellular reactions. In such a way, HPV genome replication could be favored by the Warburg effect, which would provide the urgent needs of ATP and nucleotides, required in different steps of the viral life cycle.

## 7. Metabolism as a Therapeutic Target in HPV-Related Cancers

Oncologic patients undergo tumoral chemo- and radioresistance, due in part to the hypoxic tumor microenvironment. It has been shown that aerobic glycolysis favors radioresistance [107,108]. Liu and collaborators observed that HK2 enzyme is increased in HPV-infected cervical cancer cells, compared to those negative to HPV, and that the inhibition of HK2 activity in HPV-positive cells makes these cells radiosentive [79]. Hypoxia status has been related to radioresistance in tumors and cell lines. It is known that the human epidermal growth factor receptor 2 (HER-2), a receptor tyrosine kinase (RTK), is over-expressed in HPV-related cancers; although the role of this protein remains unclear for prognosis and therapy in cervical cancer. Clinical data have shown tumor resistance to treatment to receptor tyrosine kinase inhibitors (RTK inhibitors), related to HIF1α overexpression. It has been observed that when blocking metabolic mechanisms, including hypoxia and glycolysis as adjuvant treatments, tumoral cells become sensitive to conventional drugs and radiotherapy schemes, which represents a potential strategy for cancer therapy. There is some evidence that shows that cytotoxic activity can be enhanced when combining HER inhibitors (RTKs inhibitors) with inhibitors of glucose metabolism, in relation to the use of each drug alone [109,110]. Using SiHa-derived tumors as a model for HER2 and HPV positive cervical cancer, Martinho et al. (2017) observed a poor response to HER inhibitors, associated with an increase in lactate production [111]. HER inhibitors promote a glycolytic metabolism that is related with poor response in HPV-positive cancer cells, a phenomenon that was not observed in HPV-negative tumors. Interestingly, the combination of lepatinib, receptor tyrosine kinase (RTK) inhibitor, and 2-deoxy-d-glucose (2-DG), a non-metabolizable glucose analog, decreased lactate and HIF1α expression, resulting in a decrease in cell viability, tumor growth inhibition, as well as in a decrease in angiogenesis, compared to the cytotoxic effects of both drugs alone. These results suggest the use of glycolysis inhibitors as adjuvant therapy directed to achieve an increase in the cytotoxicity of conventional chemotherapy in cancer cells [111].

Since HPV could contribute to radio- and chemoresistance by the deregulation of key metabolic pathways, it is necessary to develop new strategies, including metabolic molecular targets, to enhance the cytotoxic effects of standard chemo- or radiotherapies, which will contribute to improve clinical responses in HPV-related cancers.

## 8. Conclusions

The Warburg effect is a metabolic change observed in practically all types of cancer. In HPV-related cancers, the E6 and E7 viral oncoproteins are responsible for maintaining the malignant phenotype and promote a metabolic switch, together with other viral proteins, such as E5 and E2, even though E6 and E7 regulate two critical enzymes of the glycolytic pathway, hexokinase and pyruvate kinase, as well as other proteins involved in lactate production, such as lactate dehydrogenase. Furthermore, the E2 viral protein also stimulates the Warburg effect, decreasing the respiratory chain and oxidative phosphorylation. The Warburg effect provides a rapid production of ATP, helping to satisfy the high-energy demands of HPV-related cancer cells during cell proliferation and, probably, Warburg also contributes to HPV productive infection during the replication of the viral genome. The identification of the effects of individual HPV proteins on elements that regulate the metabolic switch, strongly suggests that HPV regulates Warburg; nevertheless, how these individual effects of HPV proteins work together on metabolic cellular pathways is still rather uncertain. Nowadays, several studies have identified new cellular interacting partners of HPV proteins [112,113,114]. The detailed analysis of some of those interactions which could possibly be involved in the regulation of the Warburg effect, as well as the study of their functional implications, will enrich the knowledge that may contribute to improve the design of new therapeutic strategies for HPV-related cancers.

## Abbreviations

HPV	Human papillomavirus
KC	Krebs cycle
OXPHOS	Oxidative phosphorylation
TIGAR	TP53 induced glycolysis and apoptosis regulator
HIF1	Hypoxia-inducible factor 1
HK2	Hexokinase 2
PK	Pyruvate kinase
PFK-1	Phosphofructokinase-1
PBM	PDZ-binding motif
HDAC1	Histone deacetylase 1
DBMT1	DNA methyltransferase
EZH2	Enhancer of zeste homolog 2
